# Aberrant expression of a stabilized β-catenin mutant in keratocytes inhibits mouse corneal epithelial stratification

**DOI:** 10.1038/s41598-018-36392-2

**Published:** 2019-02-13

**Authors:** Lingling Zhang, Yen-Chiao Wang, Yuka Okada, Suohui Zhang, Matthew Anderson, Chia-Yang Liu, Yujin Zhang

**Affiliations:** 1 School of Optometry, Indiana University, Bloomington, IN 47405 USA; 2 Department of Ophthalmology, Wakayama Medical University, Wakayama, Japan; 3 Comprehensive Cancer Center-Arthur G. James Cancer Hospital and Richard J. Solove Research Institute, School of Medicine, The Ohio State University, Columbus, OH 43210 USA

## Abstract

We previously reported that genetic deletion of β-catenin in mouse corneal keratocytes resulted in precocious corneal epithelial stratification. In this study, to strengthen the notion that corneal keratocyte-derived Wnt/β-catenin signaling regulates corneal epithelial stratification during mouse development, we examined the consequence of conditional overexpression of a stabilized β-catenin mutant (*Ctnnb1*^*ΔE3*^) in corneal keratocytes via a doxycycline (Dox)-inducible compound transgenic mouse strain. Histological analysis showed that conditional overexpression of *Ctnnb1*^*ΔE3*^ in keratocytes inhibited corneal epithelial stratification during postnatal development. Unlike the corneal epithelium of the littermate controls, which consisted of 5-6 cell layers at postnatal day 21 (P21), the mutant corneal epithelium contained 1-2 or 2-3 cell layers after Dox induction from embryonic day 0 (E0) to P21 and from E9 to P21, respectively. X-gal staining revealed that Wnt/β-catenin signaling activity was significantly elevated in the corneal keratocytes of the Dox-induced mutant mice, compared to the littermate controls. Furthermore, RT-qPCR and immunostaining data indicated that the expression of Bmp4 and ΔNp63 was downregulated in the mutant corneas, which was associated with reduced corneal epithelial proliferation in mutant epithelium, as revealed by immunofluorescent staining. However, the expression of Krt12, Krt14 and Pax6 in the mutant corneas was not altered after overexpression of *Ctnnb1*^*ΔE3*^ mutant protein in corneal keratocytes. Overall, mutant β-catenin accumulation in the corneal keratocytes inhibited corneal epithelial stratification probably through downregulation of Bmp4 and ΔNp63 in the corneal epithelium.

## Introduction

Bidirectional mesenchymal-epithelial interactions play essential roles in the development of organs with an epithelial parenchyma. Any disorder of these interactions may disrupt tissue formation and cell differentiation of both the epithelium and mesenchyme^1–3^. In mouse corneas, the outermost transparent layer of the eye serves as an ideal model for studying mesenchymal-epithelial interactions^4^. It is composed of a stratified squamous non-keratinized epithelium, a thick stroma scattered with keratocytes, and a single-layered endothelium^5^, all of which serve as a major refractive power to transmit light to the retina, as well as a protective barrier against dirt, germs and particles that can damage the eyes^6–8^.

To establish a functional cornea, complex developmental processes must be precisely coordinated by intrinsic regulators and reciprocal signal communication between the epithelium and stroma through signaling transduction, such as Wnt/β-catenin and BMP signaling pathways^9–12^. Both of these two signaling pathways play critical roles in ocular morphogenesis^13–15^. Gain and loss-of-function studies have revealed that Wnt/β-catenin signaling is involved in eye field formation, neural retina specification, and lens induction during early embryonic stages^10,16–19^. Loss of DKK2, an antagonist of the Wnt/β-catenin signaling pathway, suppresses corneal differentiation during mouse development^20,21^. Ectopic expression of *Ctnnb1*^*ΔE3*^ in corneal epithelial cells leads to corneal intraepithelial neoplasia^22^, which implies that Wnt/β-catenin signaling in the corneal epithelium needs to be repressed during embryonic development and adult homeostasis. BMP4 signaling is involved in cell differentiation and lens induction^13,23^. Crosstalk between Wnt/β-catenin and BMP4 signaling has been observed in multiple developmental events^9,12,24–28^. However, the roles of Wnt/β-catenin and BMP4 signaling pathways and signal crosstalk between them during corneal development are largely unknown, and the mechanism by which corneal keratocyte-derived signals contribute to these processes in the cornea has yet to be fully elucidated.

Recently, we reported that conditional disruption of Wnt/β-catenin signaling by deletion of its key mediator, β-catenin(*Ctnnb1*^*cKO*^) or co-receptor Lrp5 and Lrp6 (*Lrp5*^*cKO*^ & *Lrp6*^*cKO*^), in mouse corneal keratocytes results in precocious corneal epithelial stratification^9^. In this study, taking advantage of the gain-of-function strategy, we found that expression of a stabilized β-catenin mutant, *Ctnnb1*^*ΔE3*^, in corneal keratocytes inhibited corneal epithelial stratification, an exactly opposite phenotype caused by β-catenin deletion in stromal cells during development. Interestingly, the protein levels of *Bmp4* and *ΔNp63* were downregulated in the cornea after expression of *Ctnnb1*^*ΔE3*^, which may be responsible for the inhibition of corneal epithelial stratification. Collectively, our data indicated that corneal keratocyte-derived Wnt/β-catenin signaling plays indispensable roles in corneal epithelial maturation during mouse ocular surface development.

## Results

### Expression of *Ctnnb1*^*ΔE3*^ in keratocytes inhibited mouse corneal epithelial stratification

**Figure 1:**
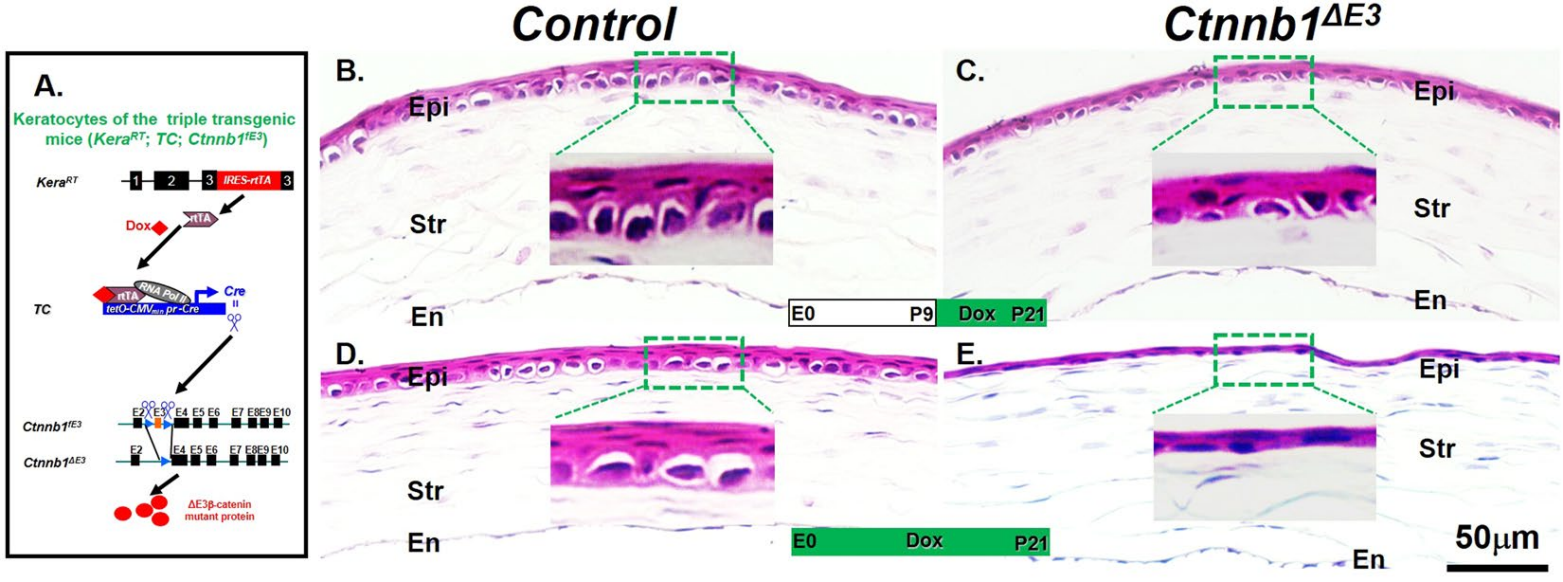
Corneal epithelial stratification was inhibited in the *Ctnnb1*^*ΔE3*^ mutant mice after Dox induction. (**A**) Schematic representation of conditional expression of a stabilized β-catenin mutant (*Ctnnb1*^*ΔE3*^) in the corneal keratocytes of the triple transgenic mice (*Kera*^*RT*^; *TC*; *Ctnnb1*^*fE3*^) after Dox induction. (**B**–**E**) H&E stain showed that the corneal epithelium had 5-6 stratified cell layers in littermate controls (**B**,**D**). In contrast, the *Ctnnb1*^*ΔE3*^ mutant corneal epithelium consisted of 2-3 and 1-2 cell layers (**C**,**E**) when Dox-induced from P9 to P21 (compare **B**–**C**) and E0-P21 (compare **D**,**E**), respectively. Abbreviations: Epi: corneal epithelium; Str, stroma; En, endothelium.

Previously, we reported that deletion of β-catenin, specifically in keratocytes of the triple transgenic mice (*KR*^29^; *TC*^30^; *Ctnnb1*^*flox/flox*^ ^31^), resulted in precocious corneal epithelial stratification during morphogenesis and postnatal development^9^. This interesting phenotype prompted us to investigate whether ectopic overexpression of a stabilized β-catenin mutant, *Ctnnb1*^*ΔE3*^, in mouse keratocytes has any effect on corneal epithelial stratification during development. To this end, a new triple transgenic mouse strain (*Kera*^*RT*^ ^32^; *TC*; *Ctnnb1*^*fE3*^ ^33^) was generated to express Ctnnb1^ΔE3^ mutant protein (ΔE3β-catenin) in corneal keratocytes upon Dox administration. We administered Dox from E0 to P21 and from P9 to P21, respectively. Like the littermate controls, the *Ctnnb1*^*ΔE3*^ mutant mice were able to develop clear and transparent eyes (data not shown). However, hematoxylin and eosin (H&E) stain showed that, instead of forming 5-6 stratified corneal epithelial cell layers in the littermate controls at P21, expression of *Ctnnb1*^*ΔE3*^ in keratocytes resulted in forming significant thinner corneal epithelia ranging from 1 to 3 cell layers, depending on the time of Dox administration. (Fig. 1B–E). We also found a more profound effect on corneal epithelial stratification when *Ctnnb1*^*ΔE3*^ was aberrantly expressed during embryonic development, as compared to that with Dox induction during postnatal development (compare Fig. 1B–E). These data suggest that corneal epithelial stratification was inhibited by expression of *Ctnnb1*^*ΔE3*^ in the corneal keratocytes during development.

### Expression of *Ctnnb1*^*ΔE3*^ in keratocytes enhanced canonical Wnt signaling activity in mouse corneal stroma

**Figure 2:**
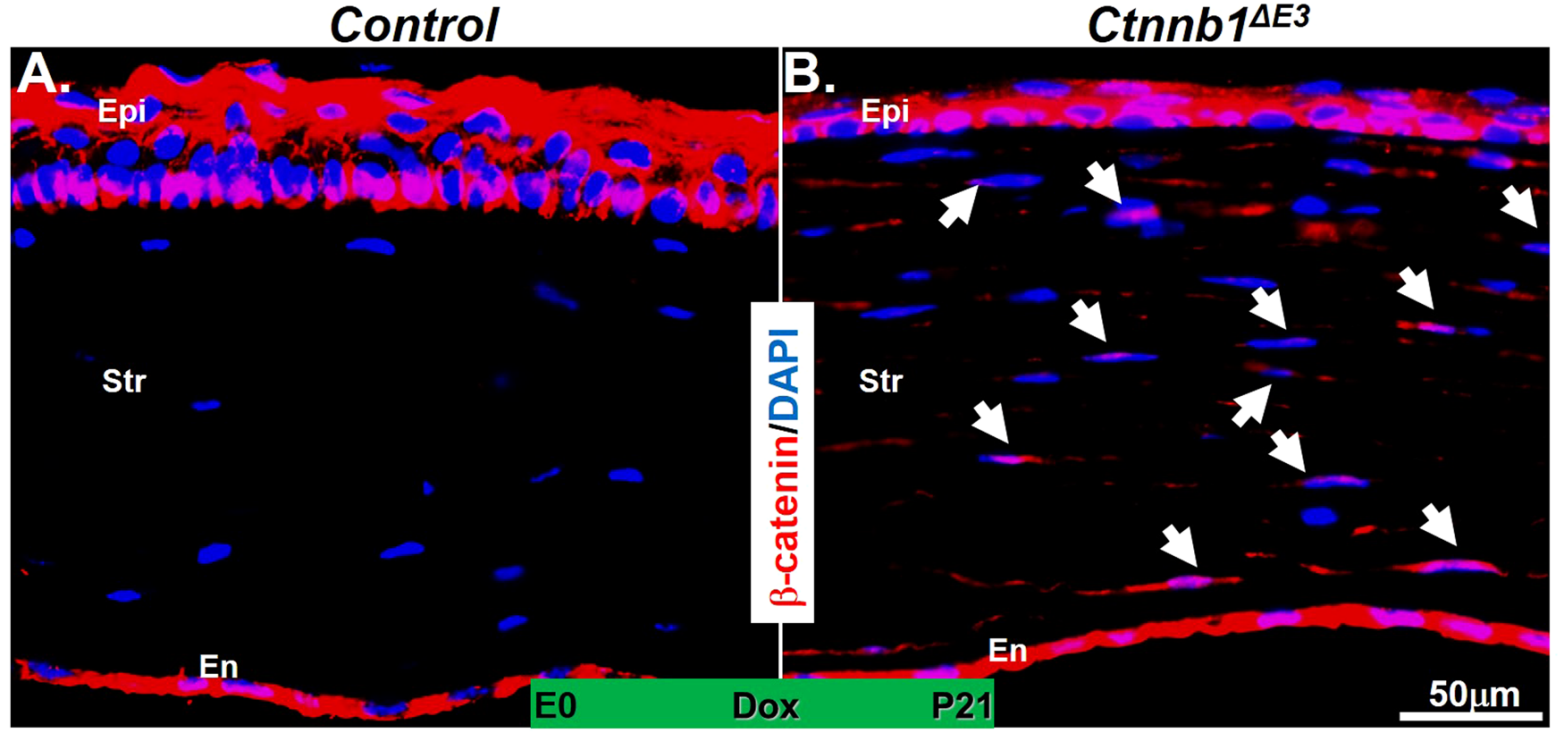
Immunofluorescent staining indicated that β-catenin was translocated to the nucleus in the corneal stroma of the *Ctnnb1*^*ΔE3*^ mutant mice. Nuclear-localized β-catenin was not detected in the corneal stroma of littermate controls (**A**). In contrast, strong signals were discovered in the *Ctnnb1*^*ΔE3*^ corneal stroma (arrows in **B**).

To confirm that the inhibition of corneal epithelial stratification in *Ctnnb1*^*ΔE3*^ mutant mice was due to the expression of *Ctnnb1*^*ΔE3*^ in corneal keratocytes, immunofluorescent staining probed with anti-β-catenin antibody was performed. We found that β-catenin was observed abundantly in epithelium and endothelium of both mutant and littermate controls. However, β-catenin was hardly detected in the keratocytes of the littermate controls (Fig. 2A). In contrast, β-catenin with some nuclear translocation was intensely observed in the keratocytes of *Ctnnb1*^*ΔE3*^ mutant mice (Fig. 2B), which implies that canonical Wnt signaling was activated in mutant corneal keratocytes after *Ctnnb1*^*ΔE3*^ expression. Taken together, these data suggest that the expression of ΔE3β-catenin in the keratocytes inhibited corneal epithelial stratification during development.

**Figure 3:**
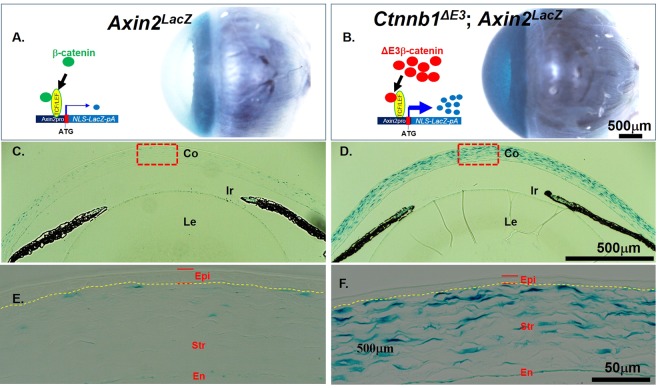
X-gal staining revealed that Wnt/β-catenin signaling activity is extremely elevated in corneal stroma after expression of *Ctnnb1*^*ΔE3*^ in corneal keratocytes. (**A**) Schematic representation of turning on the *lacZ* gene by β-catenin in corneal keratocytes of the *Axin2*^*LacZ*^ knock-in mouse line (left) and whole mount X-gal staining of enucleated eyeball (right) from *Axin2*^*LacZ*^ mice. (**B**) Schematic representation of turning on the *lacZ* gene by the stabilized ΔE3b-catenin mutant protein in corneal keratocytes of the compound transgenic mice (*Kera*^*RT*^; *TC*; *Ctnnb1*^*fE3*^; *Axin2*^*LacZ*^) after Dox induction (left) and whole mount X-gal staining (dark blue) of enucleated eyeball (right) from these quadruple transgenic mice (n = 4). (**C**,**D**) Paraffin sections of X-gal stained eyeballs of *Axin2*^*LacZ*^ (**C**) and quadruple transgenic mice (**D**). (**E**,**F**) Higher magnification photographs of C and D in the dotted boxes to show X-gal staining in the central corneas. Note that strong X-gal staining was localized in the corneal stroma of the quadruple transgenic eyeball (**D**,**F**), while only a very few of keratocytes in *Axin2*^*LacZ*^ showed positive signals (**E**). The yellow dashed line in E and F delineates the corneal epithelium layers. The red double short lines in E and F indicate the corneal epithelium thickness. Abbreviations: Co, cornea; Ir, Iris.

To further confirm that nuclear localized mutant ΔE3β-catenin protein is able to activate the expression of a downstream target gene, such as *Axin2*, a typical target gene of canonical Wnt signaling, the quadruple transgenic mouse strain, *Kera*^*RT*^; *TC*; *Ctnnb1*^*fE3*^; *Axin2*^*LacZ*^ ^34^ was generated by mating the knock-in mouse line, *Axin2*^*LacZ*^, with the triple transgenic mouse strain *Kera*^*RT*^; *TC*; *Ctnnb1*^*fE3*^. X-gal staining showed that strong dark blue signals were detected only in the corneas of the quadruple transgenic mice administered Dox chow from P9 to P21, but very faint blue appeared in the *Axin2*^*LacZ*^ littermate controls (Sup. Fig.1, compare the right panel of Fig. 3B to the left panel of 3A). Histological examination revealed that X-gal staining was detected specifically in the corneal stroma of the quadruple transgenic mice (Fig. 3D,F). In contrast, much fainter blue signals in *Axin2*^*LacZ*^ littermate controls were spotted in only a few of the corneal keratocytes (Fig. 3C,E). These data demonstrate that canonical Wnt signaling activity is low in normal corneal keratocytes at P21^9,22^. Ectopic overexpression of *Ctnnb1*^*ΔE3*^ mutant significantly enhanced the expression of downstream targets of the canonical Wnt signaling pathway. Collectively, activation of canonical Wnt signaling by expression of *Ctnnb1*^*ΔE3*^ in corneal keratocytes caused the inhibition of corneal epithelial stratification in the mutant mice.

### Expression of *Ctnnb1*^*ΔE3*^ in keratocytes decreased epithelial cell proliferation, but did not change the differentiation status of the corneal epithelium

**Figure 4:**
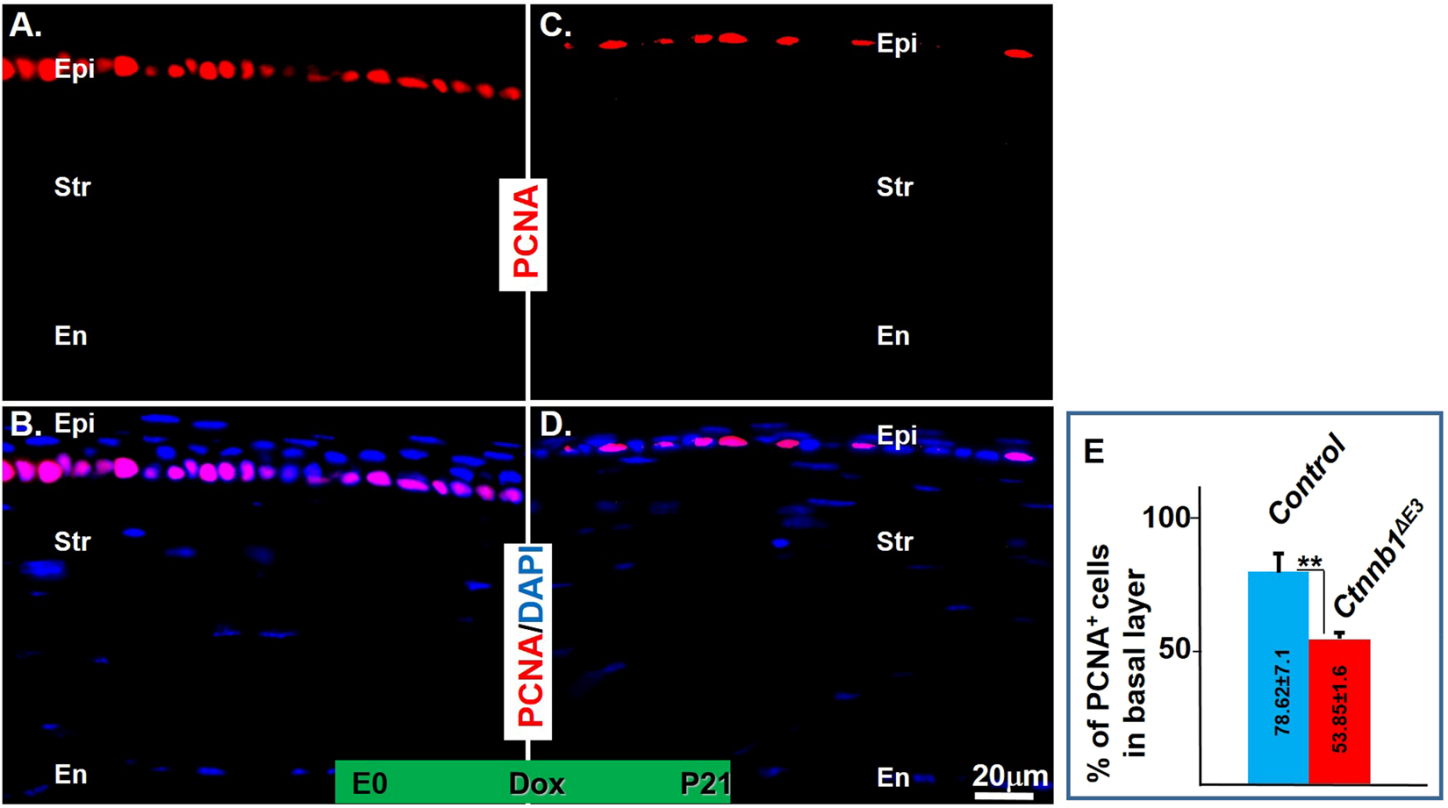
Immunofluorescent staining showed PCNA expression was significantly decreased in the basal corneal epithelium of the *Ctnnb1*^*ΔE3*^ mutant mice. PCNA expression was detected in basal corneal epithelial cells of littermate controls at P21 (**A**,**B**). Note that PCNA expression is down-regulated in basal epithelial cells of *Ctnnb1*^*ΔE3*^ mutant mice. (**C**,**D**). (**E**) Quantitative analysis showed the percentage of PCNA-positive basal cells in *Ctnnb1*^*ΔE3*^ corneas was dramatically decreased compared to controls, n = 4, **P < 0.01. Mean ± s.e.m.

Canonical Wnt signaling plays fundamental roles in controlling cell fate, differentiation, proliferation, and apoptosis during development^16,35^. Inhibition of corneal epithelial stratification in *Ctnnb1*^*ΔE3*^ mutant mice may be attributed to either slower proliferation rates or increased cell death. Results of the TUNEL assay did not show a difference between the littermate controls and mutant mice (data not shown). To detect any changes in corneal epithelial cell proliferation between littermate controls and mutant mice, the expression of PCNA was examined by immunofluorescent staining. The data showed that the percentage of PCNA-positive cells in the basal corneal epithelium of *Ctnnb1*^*ΔE3*^ mice was dramatically reduced to 53.85%, compared to 78.85% in littermate controls (Fig. 4E; compare Fig. 4A–D). Likewise, immunofluorescent staining against Ki67, another proliferation marker, revealed that positive signals in mutant epithelium were also dramatically reduced, compared to that in littermate controls (Fig. 1C; compare Sup. Fig. 2AB). These data indicate that expression of *Ctnnb1*^*ΔE3*^ in mouse keratocytes reduced corneal epithelial cell proliferation during postnatal development, which may account for the inhibition of epithelial stratification in the mutant mice.

**Figure 5:**
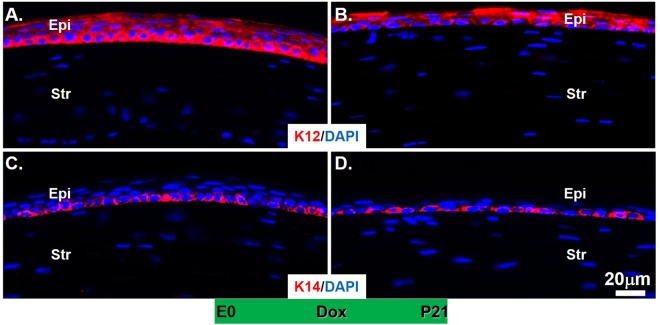
Immunofluorescent staining showed that the expression of Krt12 and Krt14 was maintained in the corneal epithelium of the *Ctnnb1*^*ΔE3*^ mutant mice. (**A**,**B**) The central corneal epithelium in littermate controls (**A**) and mutants (**B**) displayed strong expression of Krt12 at P21. (**C**,**D**) Immunofluorescent staining indicated that Krt14 expression in basal corneal epithelium of *Ctnnb1*^*ΔE3*^ was not altered after expression of *Ctnnb1*^*ΔE3*^ mutant in corneal keratocytes, n = 4.

**Figure 6:**
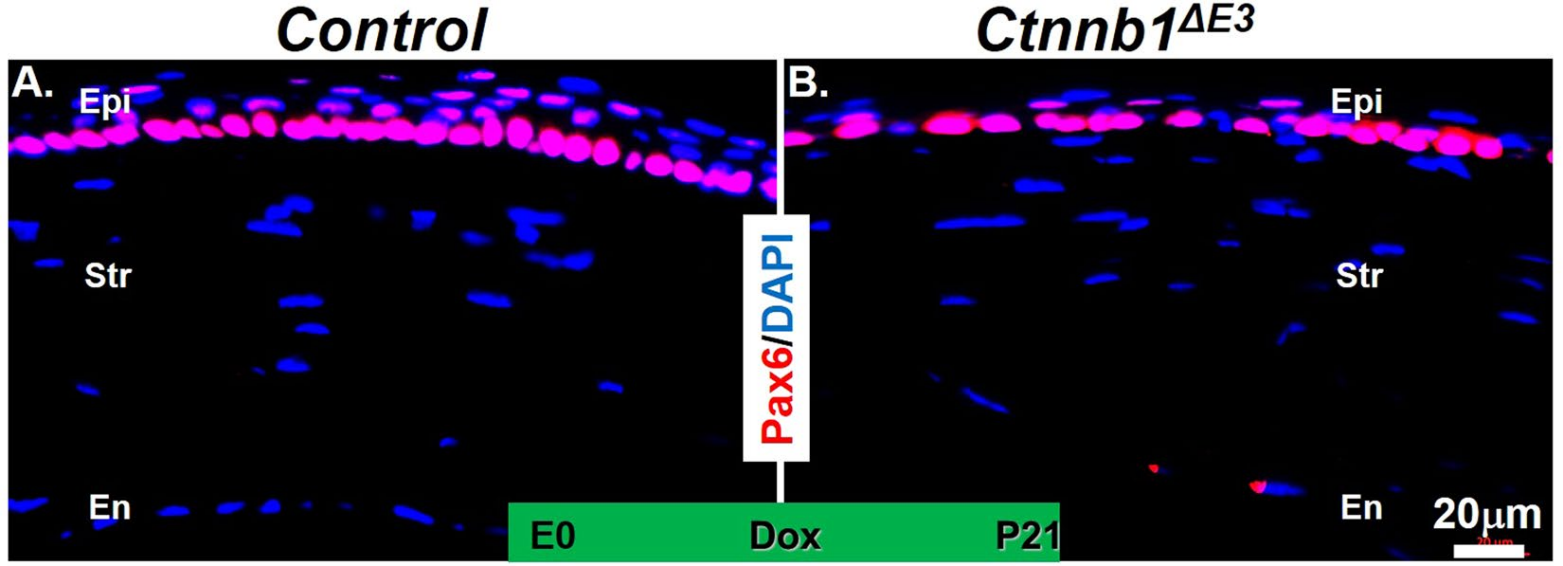
Pax6 expression was not altered in the *Ctnnb1*^*ΔE3*^ mutant corneal basal epithelium. (**A**,**B**) Immunofluorescent staining showed that Pax6 expression in the corneal epithelium of *Ctnnb1*^*ΔE3*^ was maintained after overexpression of the mutant *Ctnnb1*^*ΔE3*^ in corneal keratocytes (**B**) compared with littermate controls (**A**), n = 4.

We previously reported that expression of *Ctnnb1*^*ΔE3*^ in the corneal epithelium of the triple transgenic mice (*K12*^*rt*^; *TC*; *Ctnnb1*^*fE3*^) caused corneal epithelium hyperplastic transformation, which also displayed loss of Krt12 and Pax6 expression in the corneal epithelium^22^. In this study, we asked whether expression of *Ctnnb1*^*ΔE3*^ in corneal keratocytes could influence corneal epithelial differentiation. Our data show that Krt12 expression in both mutant and littermate controls was restricted to the corneal epithelium, but was not found in the stroma (Fig. 5A,B). Likewise, Krt14 expression was detected in the basal corneal epithelia of both the mutant and controls (Fig. 5C,D). Furthermore, immunofluorescent staining showed that Pax6 expression was detected in the corneal epithelium of both mutants and the littermate controls (Fig. 6). These data suggest that the characteristic features of corneal epithelium remained, despite the inhibition of corneal epithelial stratification in the *Ctnnb1*^*ΔE3*^ mutant mice.

### Expression of Bmp4 and ΔNp63 was decreased in *Ctnnb1*^*ΔE3*^ mutant mice corneas

Given that genetic ablation of *Ctnnb1* in mouse corneal keratocytes resulted in upregulation of Bmp4 expression^9^, which in turn acted as a paracrine growth factor, triggering corneal epithelial stratification via upregulation of a transcription factor, p63. To investigate whether Bmp4 is involved in the inhibition of corneal epithelial stratification in *Ctnnb1*^*ΔE3*^ mutant mice, we first examined the Bmp4 expression pattern in wild-type mouse corneas during development. Both real-time quantitative PCR (RT-qPCR) and regular reverse-transcription PCR (RT-PCR) showed that, at P10 and P21, Bmp4 expression was much stronger in the stroma than in the corneal epithelium (Sup. Fig. 3). It is interesting to note that the Bmp4 expression ratio of corneal stroma to epithelium increased from P10 to P21 (Sup. Fig. 3). These data suggest that the stroma is the main source of Bmp4 in the cornea during its epithelial stratification.

**Figure 7:**
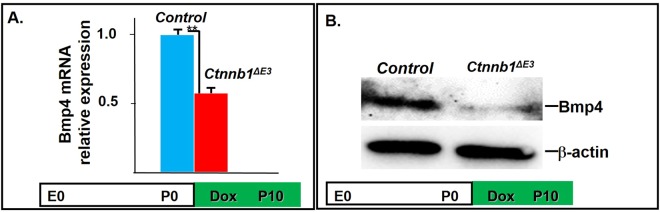
Bmp4 expression was decreased at protein and mRNA levels in mutant *Ctnnb1*^*ΔE3*^ corneas. (**A**) RT-qPCR indicating *Bmp4* mRNA was decreased in *Ctnnb1*^*ΔE3*^ mutant corneal stroma with Dox induction from P0 to P10, n = 4, **P < 0.01. Mean ± s.e.m. (**B**) Western blot results confirmed Bmp4 protein was significantly reduced in *Ctnnb1*^*ΔE3*^ mice, n = 3.

**Figure 8:**
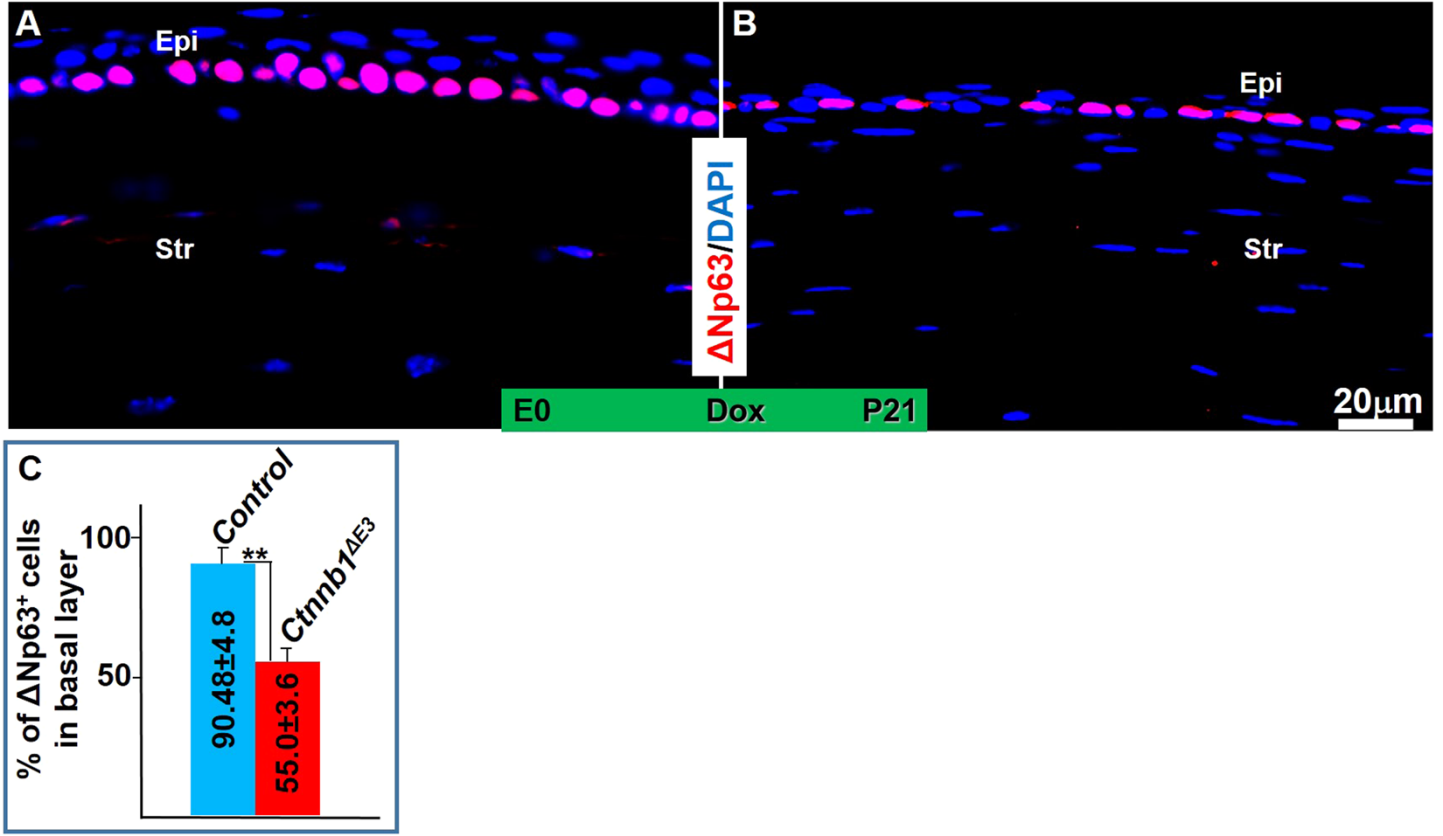
ΔNp63 expression was down-regulated in the *Ctnnb1*^*ΔE3*^ mutant corneal basal epithelium. (**A**,**B**) Immunostaining showed that ΔNp63 expression in corneal epithelium of *Ctnnb1*^*ΔE3*^ was reduced (**B**) compared with littermate controls (**A**). (**C**) Quantitative analysis showed the percentage of ΔNp63-positive basal cells in *Ctnnb1*^*ΔE3*^ corneas was significantly decreased compared to controls, n = 4, **P < 0.01. Mean ± s.e.m.

Based on our previous report^9^, we hypothesized that Bmp4 may be associated with the inhibition of corneal epithelial stratification in *Ctnnb1*^*ΔE3*^ mutant mice. As expected, RT-qPCR revealed that Bmp4 expression in corneal stroma was dramatically reduced in *Ctnnb1*^*ΔE3*^ mutant mice with Dox induction from P0 to P10 (Fig. 7A). Accordingly, expression of Bmp4 at the translational level was also downregulated in *Ctnnb1*^*ΔE3*^ mutant corneal stromal cells (Fig. 7B). More interestingly, the expression of ΔNp63, the main isoform of TP63, expressed in basal corneal epithelial cells, was downregulated in mutant corneas (Fig. 8A–C). Considering that canonical Wnt signaling suppressed Bmp4 expression in corneal stroma and Bmp4 regulated p63 expression in corneal epithelium during postnatal development^9^, current data further validate our hypothesis that aberrant activation of canonical Wnt signaling in corneal keratocytes represses Bmp4 expression, leading to the down-regulation of p63 expression and inhibition of corneal epithelial stratification.

## Discussion

In this study, we discovered that a stabilized β-catenin mutant, *Ctnnb1*^*ΔE3*^, aberrantly expressed in corneal keratocytes by means of a Dox-inducible compound transgenic mouse model (*Kera*^*RT*^; TC; *Ctnnb1*^*fE3*^) caused the inhibition of corneal epithelial stratification during ocular surface postnatal morphogenesis, which may be attributed to decreased proliferation of the basal corneal epithelial cells, caused by down-regulation of the expression of Bmp4 and ΔNp63 in the mutant mice. This phenotype is the exact opposite of the precocious corneal epithelial stratification observed in the β-catenin loss of function mutant mice (*Ctnnb1*^*cKO*^), as we reported previously^9^. Notably, we found that both Bmp4 and ΔNp63 were involved in these opposite phenotypes in corneal epithelium. Based on the data from the gain-of-function model, in this study, and loss-of-function data previously reported, we propose that during mouse corneal postnatal morphogenesis, stromal cell-derived canonical Wnt signaling negatively modulates the expression of Bmp4, which, in turn, functions as a paracrine growth factor to control corneal epithelial stratification via the transcriptional factor, p63. In this case, the expression of a stabilized mutant of β-catenin in stromal cells inhibited the expression of Bmp4, which would lead to decreased corneal epithelial cell proliferation and inhibition of epithelial stratification due to decreased ΔNp63 expression in the basal epithelial cells (Figs 4, 7 and 8). This is a novel example of the signaling interaction between mouse corneal stroma and epithelium, one of the most important mechanisms by which a functional mouse cornea is properly developed during postnatal morphogenesis.

Soluble growth factors and cytokines are generally considered to be the central mediators, which transmit signals between the stroma and epithelium in bidirectional communication^2,36^. Hepatocyte growth factor (HGF), keratocyte growth factor (KGF), epidermal growth factor (EGF), and transforming growth factor alpha (TGF-a) have been characterized as the growth factors produced in corneal stroma and have intense impacts on epithelium morphogenesis by regulating its proliferation, motility, and differentiation^2,4^. Other growth factors and cytokines from the stroma may also contribute to these interactions during development, homeostasis, and wound healing of the cornea. In the current study, we discovered that corneal stromal cells were the main source of Bmp4 produced in corneas during development. Furthermore, we took advantage of two Dox-inducible compound transgenic mouse strains (*KR*; *TC*; *Ctnnb1*^*flox/flox*^ and *Kera*^*RT*^; *TC*; *Ctnnb1*^*fE3*^) to genetically manipulate β-catenin expression, specifically in corneal keratocytes, and successfully identified Bmp4 as a new mediator, derived from stromal cells to modulate corneal epithelial stratification during embryonic development and postnatal morphogenesis. Notably, our study also provided genetic evidence showing that Bmp4 expression in stromal cells is negatively regulated by canonical Wnt signaling during corneal postnatal morphogenesis^9^. Therefore, the interplay between canonical Wnt signaling and Bmp4 signaling may form the switch to coordinately control corneal epithelial stratification after mouse birth to establish multiple, well organized cell layers with proper function before eyelid opening around P12 to P14.

Canonical Wnt signaling plays vital roles in ocular morphogenesis, homeostasis, wound healing, and pathogenesis of many ocular diseases^10,13–15,37–39^. However, to the best of our knowledge, most of the genetic studies examining the role of canonical Wnt signaling in mouse corneas were performed with corneal epithelium^21,22,40–42^. For example, it was documented that repression of canonical Wnt signaling activity in corneal epithelium is an essential prerequisite for the differentiation of corneal epithelial cells from their progenitors, as reported in the *Dkk2* knockout mice^21^ and in *Pitx2*-deficient mice^20^. Similarly, canonical Wnt signaling should be stringently controlled in corneal epithelium for the proper maintenance of normal corneal structure and function at the mouse adult stage because expression of a stable mutant form of β-catenin (*Ctnnb1*^*ΔE3*^) in adult corneal epithelial cells led to corneal intraepithelial neoplasia^22^. We made use of the keratocan (*Kera*) gene promoter/enhancer region to drive a reverse tetracycline transactivator (rtTA) and generated the Dox-inducible mouse lines, *KR* and *Kera*^*RT*^ by transgenic and knock-in strategies, respectively. These two strains can be used to manipulate gene expression in corneal keratocytes since *kera* gene is exclusively expressed in the neural crest derived cells, including corneal keratocytes^9,29,43^. Benefitting from these two special driver mouse lines, we knocked out β-catenin (*Ctnnb1*^*cKO*^) previously^9^ and ectopically expressed a stable mutant form of β-catenin (*Ctnnb1*^*ΔE3*^) in the present study, specifically in corneal keratocytes. Excitingly, we observed opposite corneal epithelium phenotypes from these two mutant mice after modifying β-catenin genetically in corneal keratocytes. Additionally, the same or similar molecular mechanism, by which canonical Wnt signaling impacts corneal epithelial maturation, may be implicated in these phenomena because the same molecules, such as Bmp4 and ΔNp63, were involved. In addition to the inhibition of corneal epithelial stratification, we noticed that the expression of corneal stromal specific markers, *Lum* and *Kera*, in mutant stroma was reduced after *Ctnnb1*^*ΔE3*^ expression (data not shown), which may imply that the differentiation status of corneal keratocytes had been altered after expression of *Ctnnb1*^*ΔE3*^. In view of the changes in the components of the TGFβ signaling pathway in the *Ctnnb1*^*cKO*^ mice^9^, we propose that reduced expression of *Lum* and *Kera* might be attributed to the changes in TGFβ signaling activity. The exact molecular mechanism is being investigated in our laboratory.

Bmp4 was previously ascertained as the key mediator linking the corneal epithelial phenotype, instigated by loss of β-catenin. However, we did not exclude other growth factors and cytokines which may contribute to the phenotype, since changes in the expression of several growth factors, including FGF family members, were detected in the *Ctnnb1*^*cKO*^ mice^9^. Therefore, we are currently investigating the possible roles of the FGF signaling pathway in the *Ctnnb1*^*ΔE3*^ mutant mice, besides Bmp4 as the first priority candidate, which may both be regulated by canonical Wnt signaling in corneal keratocytes and in turn may mediate corneal epithelial stratification in a paracrine manner.

In summary, aberrant expression of the stabilized β-catenin mutant, *Ctnnb1*^*ΔE3*^ in mouse corneal keratocytes inhibits corneal epithelial stratification. Bmp4 and ΔNp63 may be responsible for the inhibition of epithelial stratification caused by *Ctnnb1*^*ΔE3*^. Our data suggest that corneal keratocyte-derived Wnt/β-catenin signaling plays indispensable roles in corneal epithelium maturation during mouse ocular surface development.

## Materials and Methods

### Mouse strains and genotyping

All of the genetically modified mouse lines, *Kera*^*RT*^ ^32^, *TetO-Cre* (*TC)*^30^, *Ctnnb1*^*fE3*^ ^33^ and *Axin2*^*LacZ*^ ^34^ have been previously described. Compound transgenic mice were generated via natural mating of each individual mouse line. All the mice were bred at the Animal Facility of the School of Optometry, Indiana University. Experimental procedures for handling the mice were approved by the Institutional Animal Care and Use Committee, Indiana University. Animal care and use are conformed to the ARVO Statement for the Use of Animals in Ophthalmic and Vision Research. The identification of each transgenic allele was performed by polymerase chain reaction (PCR) using tail genomic DNA as templates. Primer pairs used in genotyping are summarized in Supplementary Table 1.

### Administration of Dox chow

Mice were subjected to systemic induction by Dox chow (1 g/Kg, Custom Animal Diets, Bangor, PA). To induce mice from the embryonic stage, pregnant dams were given an intraperitoneal (IP) injection of doxycycline (Dox; 80 μg/g body weight in phosphate buffered saline (PBS), pH7.4; Clontech Laboratories), then fed Dox chow (*ad libitum*). Control animals were littermates with either single or double transgene(s).

### X-gal staining for detection of β-galactosidase activity

Excised eyes were fixed in 4% PFA/PBS (paraformaldehyde in PBS pH7.4) for 30 min at 4 °C, then incubated in X-gal staining solution (5 mM potassium ferricyanide, 5 mM potassium ferrocyanide, 2 mM MgCl_2_, 0.02% NP-40, 0.01% sodium deoxycholate, 0.4 mg/ml X-gal in PBS buffer) overnight at room temperature followed by post-fixation in 4% PFA/PBS at 4 °C overnight. Whole mount and paraffin sections were subjected to examination via a stereomicroscope (EVOSFL Auto, life technologies).

### Hematoxylin and Eosin (H&E) stain and immunofluorescent staining

Enucleated eyes were fixed overnight in 4% PFA in PBS at 4 °C, followed by dehydration and paraffin embedding. De-paraffinized and rehydrated tissue sections (5 μm) were stained with Hematoxylin and Eosin and examined under a stereomicroscope (EVOSFL Auto, life technologies). For immunofluorescent staining, tissue sections were de-paraffinized, rehydrated and subjected to antigen retrieval in sodium citrate buffer (10 mM sodium citrate, 0.05% Tween-20, pH 6.0) at boiling temperature for 30 minutes. Corneal sections were then blocked with 3% bovine serum albumin (BSA) in PBS containing 0.05% NP-40 for 1 hour at room temperature, then incubated overnight at 4 °C with the primary antibodies diluted in the same buffer. After three washes in PBST (PBS/0.1% Tween-20), slides were incubated at room temperature for 1 hour with Alexa Fluor 488- or Alexa 555-conjugated secondary antibodies (Invitrogen) and 1 μg/ml DAPI (Cat: #D3571; Molecular Probes, Inc. Eugene, OR) as a nuclear counterstain, washed with PBST again, and mounted with Mowiol (Sanofi-Aventis U.S.). Sections were photographed using a Zeiss microscope equipped with a camera (Axiocam Mrm). For data acquisition, we used the Axiovision 4.6 software (Carl Zeiss). The specifications for the antibodies used in this study are listed in supplementary Table 2.

### Real-time quantitative PCR (RT-PCR) and reverse transcription PCR (RT-PCR)

Corneal epithelial cells were scrapped and removed under Zeiss stereo microscope by using a rotating burr (Algerbrush II) from two ketamine/xylazine-anesthetized compound transgenic mice (*Kera*^*RT*^; *TC*; *Ctnnb1*^*fE3*^; *TH2-GFP*^44^ as experimental mice) and two double transgenic mice (*Kera*^*RT*^; *TH2-GFP* as control), which were Dox-induced from P0 to P10. The left stromal cells with GFP green fluorescence were collected under stereo microscope and stored at −80 °C until used. Total RNA (10 μg) was isolated from the mouse corneal stroma using Trizol reagent (Invitrogen), then annealed to random primers and reverse transcribed with avian reverse transcriptase (RT) kits (Promega), according to the manufacturer’s instructions. RT-PCR was performed using C1000Touch Thermal cycler (Bio-Rad Laboratories Inc.). 30–35 PCR cyclers were carried out to detect the expression of Bmp4 and housekeeping gene Gapdh. RT-qPCR was performed using the CFX96 real-time system equipped with a C1000™ Thermal Cycler (Bio-Rad Laboratories Inc.). After the initial 3 minute denaturing step at 95 °C, 40 subsequent cycles at 95 °C lasting 15 seconds, 62 °C for 15 seconds, and 72 °C for 20 seconds were performed. The cycle threshold values were used to calculate the normalized expression of genes of interest against *Gapdh* using Q-Gene software. Primer pairs are listed in Supplementary Table 1.

### Western blotting analysis

Corneal stromal tissues without epithelium were collected as mentioned in the above RT-qPCR methods. Frozen samples were homogenized in RIPA buffer (50 mM Tris base, 150 mM NaCl, 0.5% deoxycholic acid-sodium salt, 2% SDS, and 1% NP40, pH 7.5) containing 1x protease inhibitor cocktail (Sigma P8340). Cell lysates (20 µg) from each sample were separated on a 4–20% linear gradient Tris-HCl denaturing polyacrylamide Ready Gel (Bio-Rad) and transferred to PVDF membrane (Whatman). Membranes were blocked with 5% nonfat milk in TBST (10 mM Tris-HCl pH 8.0, 150 mM NaCl, 0.05% Tween 20) and probed with primary antibody in the same buffer overnight at 4 °C. After three washes in TBST, membranes were probed with HRP-conjugated secondary antibody for an hour at room temperature and bond second antibody was further detected using an enhanced chemiluminescence assay (Supersignal West Pico, #34080; Thermo Fisher Scientific) and examined and photographed using a VersaDoc 4000MP imaging system (Bio-Rad). Antibodies are listed in Supplementary Material Table S2.

### Statistical analysis

A two-tailed Student’s *t*-test (Excel, Microsoft, Redmond, WA, USA) was used to analyze the significance of difference; *P* < 0.05* was considered statistically significant and *P* < 0.01** was considered highly statistically significant.

## Electronic supplementary material

supplementary information
