# Supplementary material for: Aberrant expression of a stabilized β-catenin mutant in keratocytes inhibits mouse corneal epithelial stratification
Source: Sci Rep. 2019 Feb 13;9:1919. doi: 10.1038/s41598-018-36392-2 (PMC6374483; doi:10.1038/s41598-018-36392-2)

Supplementary Information

Aberrant expression of a stabilized β-catenin mutant in keratocytes inhibits mouse corneal epithelial stratification

Lingling Zhang[1], Yen-Chiao Wang[1], Yuka Okada [1, 2], Suohui Zhang[3], Matthew Anderson[1], Chia-Yang Liu[*1], Yujin Zhang[*1]

[1] School of Optometry, Indiana University, Bloomington, IN 47405, USA

[2] Department of Ophthalmology, Wakayama Medical University, Wakayama, Japan

[3] Comprehensive Cancer Center-Arthur G. James Cancer Hospital and Richard J. Solove

Research Institute, School of Medicine, The Ohio State University, Columbus, OH 43210, USA

[*]Correspondence: Chia-Yang Liu (liuchia@iu.edeu), Yujin Zhang (zhang512@iu.edu) 800 E. Atwater Ave, Bloomington Indiana, 47405 Telephone: 812-856-7197

Sup figure-1 Zhang

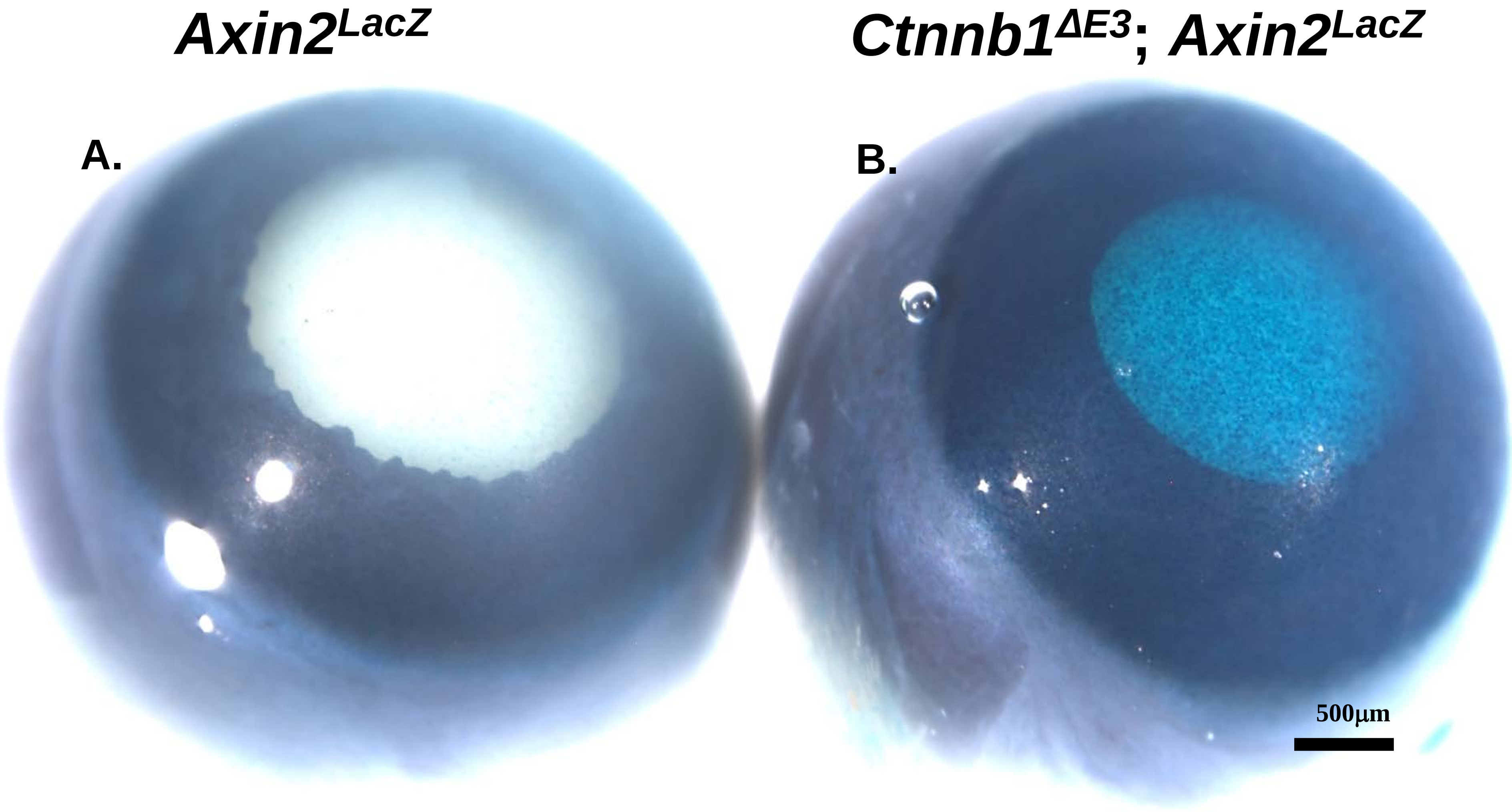

Sup figure-2 Zhang

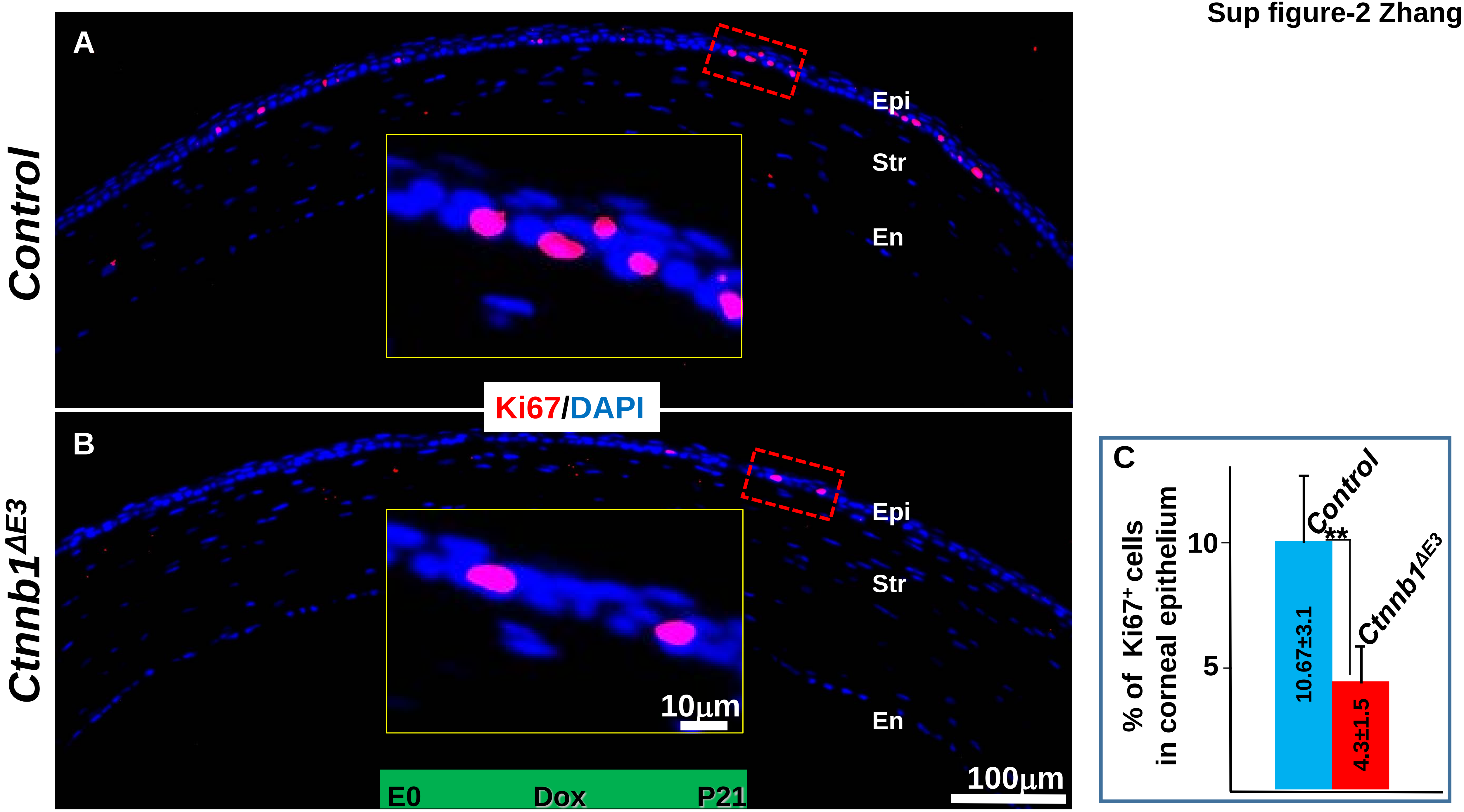

Sup figure-3 Zhang

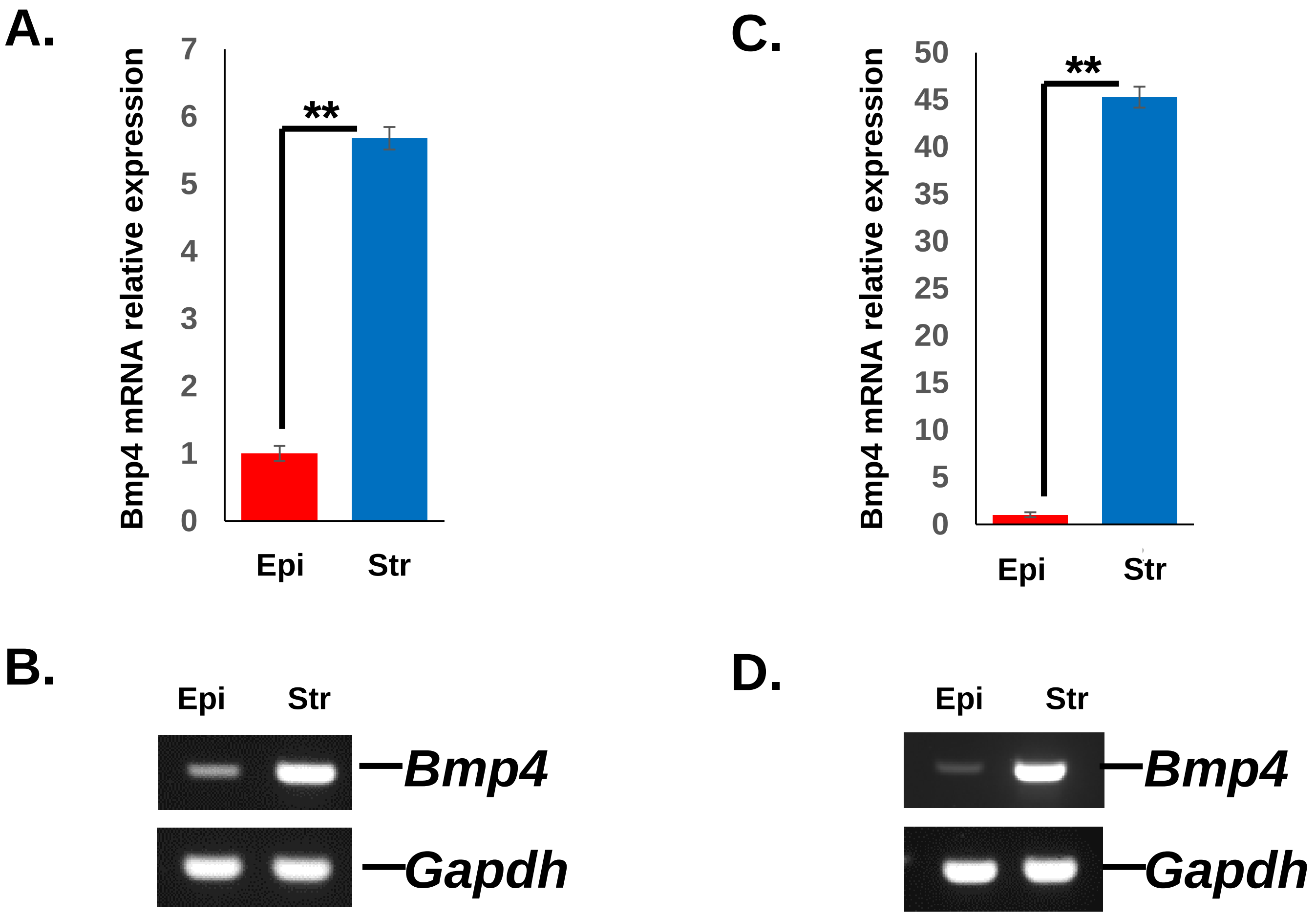

Supplementary Table S1: List of Primers used in this study

Transgenic mouse/ Gene	Sequence	Note
Kera^{RT}	5' –TGGTGGCTTGCTTCAAGCTTCTTC<br>5' –TATCCAACTCACAACGTGGCACTG<br>5' –GGAGTCTGCACTACCAGTACTCAT	mutant: 462bp<br>WT: 389bp
TC	5' –GTCAGATCGCCTGGAGACGCC<br>5' –TCGCGAACATCTTCAGGTTCTGC	only mutant produce positive PCR product (320bp)
Ctnnb1^{fE3}	5' – GACTAGTGAGACGTGCTACTT<br>5' – CGCAAGAGCAAGTAGCTGGTAA<br>5' – AGTTCCGCGTCATCCTGATAGT	mutant: 707bp<br>WT: 368bp
Axin2^{LacZ}	5' – AAGCTGCGTCGGATACTTGAGA<br>5' – AGTCCATCTTCATTCCGCCTAGC<br>5' –TGGTAATGCTGCAGTGGCTTG	mutant: 400bp<br>WT: 493bp
Bmp4	5' – CGAGCCAACACTGTGAGGAG<br>5' – CCGAGGAGATCACCTCATTC	RT-PCP for*Bmp4* expression
Gapdh	5' –AAGGTGGTGAAGCAGGCATCTGAG<br>5' –TCTTACTCCTTGGAGGCCATGTAG	RT-PCR for *Gapdh* expression

Supplementary Table S2: List of antibody used in this study

Primary antibody	Host	Source	Application
Anti-β-catenin	Mouse	#610153, BD Biosciences	IF(1ug/ml)
Anti-K12	Rabbit	custom made	IF(1:200)
Anti-K14	Mouse	#ab7800, Abcam	IF(1:200)
Anti-BMP4	Rabbit	#ab39973, Abcam	IF(1:200)
Anti-p63 (63P02)	Mouse	#MS-1082-P; Neomarkers	IF(1:200)
Anti-PCNA (PC10)	Rabbit	#ab2426, Abcam	IF(1:200)
Anti-Pax6	Rabbit	#PRB-278P, Biolegend	IF(1:200)
Anti-Ki67	Rabbit	#9129s, Abcam; Cell signaling	IF(1:200)
**Secondary antibody**			
Anti-rabbit IgG Alexa-594	Goat	Molecular Probes Ins (Eugene, OR)	IF(1:200)
Anti-Mouse IgG Alexa-594	Donkey	Molecular Probes Ins (Eugene, OR)	IF(1:200)

IF: immunohistofluorescence staining

Full-length western blots in Figure-7B

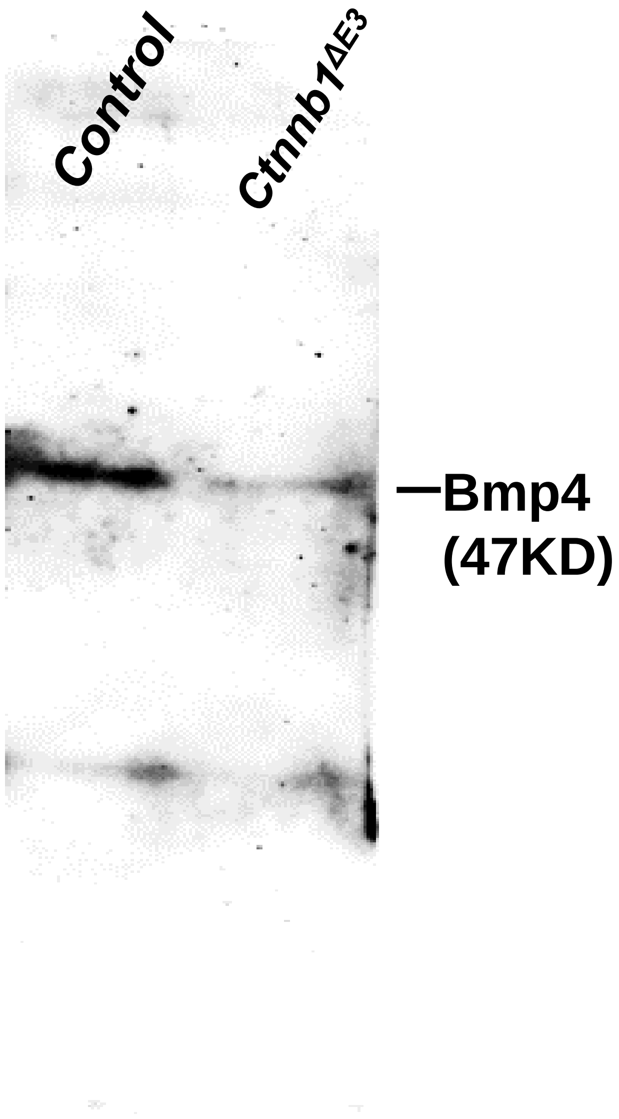

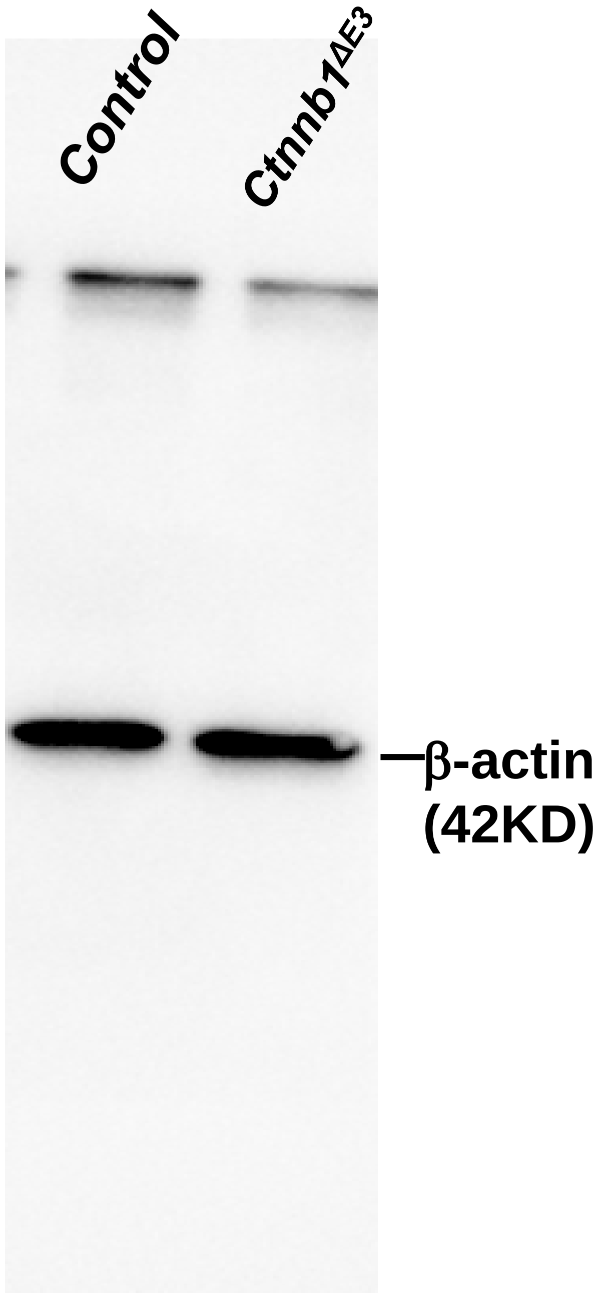

Supplement: Supplementary file 1 — supplementary information [file 41598_2018_36392_MOESM1_ESM.pdf]
